# Design of Optical Sensor Membrane Based on Polymer Poly(methyl methacrylate) for Paracetamol Detection in Traditional Herbal Medicine

**DOI:** 10.1155/2018/8918329

**Published:** 2018-06-06

**Authors:** Rimadani Pratiwi, Shelvy Elizabeth Suherman, Rajkannah A. L. Poongan, Mutakin Mutakin, Aliya Nur Hasanah

**Affiliations:** Department of Pharmaceutical Analysis and Medicinal Chemistry, Faculty of Pharmacy, Universitas Padjadjaran, Jatinangor 45363, Indonesia

## Abstract

Generally, regulation states that herbal medicines are remedies containing plants or preparation of plants as active ingredients only. Paracetamol is one of the drugs that is frequently added in herbal medicine to enhance the effect as an analgesic. The government regulation disallows chemical drugs contained in herbal medicine due to the toxic effect of uncontrolled consumption. On this study, the optical sensor membrane from polymer poly(methyl methacrylate) (PMMA) was synthesized by phase inversion method and was used to detect paracetamol in herbal medicine. PMMA was made in three different concentrations 5%, 7.5%, and 10% and was mixed with ferric chloride (FeCl3), Folin-Ciocalteu, and Nessler reagent as specific colorimetric reagents for paracetamol detection, with a ratio of solvent:reagent was 6:4; 7:3; and 8:2. The result of the experiment shows that PMMA-FeCl3 7.5% (7:3), PMMA-Folin 5% (6:4), and PMMA-Nessler 5% (6:4) give the best performance for paracetamol detection. Real herbal medicine samples were analyzed to confirm the practical application of this sensor, and the result shows good agreement with UV-Vis data. The results show that optical sensor membrane which has been developed can be used as new detection method of paracetamol for community application.

## 1. Introduction

Paracetamol is a very common analgesic and antipyretic agent since its introduction in the mid-1950s. It has been freely available without a prescription and has been commonly used for pain relief [1]. At therapeutic doses, paracetamol is effective and safe for human health. However, the abnormal level of paracetamol may cause severe diseases such as liver disorder, nephrotoxicity, and hepatic necrosis [2]. Since paracetamol can be easily obtained, it can potentially be misused. In Indonesia, paracetamol is often added to herbal medicine to enhance the therapeutic effect. Meanwhile, herbal medicines are containing plants as active ingredients only. The Indonesian Ministry of Health set the regulation for herbal medicine that it should not contain synthetic chemicals or medicinal isolation results [3]. All other countries that used herbal medicine also state that herbal medicine should not contain any synthetic chemicals. Therefore, monitoring paracetamol in herbal medicine is important to control the potential toxicity of paracetamol.

Numerous analytical methods have been developed to determine paracetamol, including spectrophotometry [4], high-performance liquid chromatography (HPLC) [5], capillary electrophoresis [6], and liquid chromatography-mass spectrometry (LC-MS) [7]. While these methods are selective and sensitive, they also require expensive instrumentation, highly trained personnel, and limited applications for on-site analysis. Recently, an electrochemical sensor for paracetamol recognition is growing [8–11]. Another interesting approach is based on the colorimetric method as a simple, rapid, and reliable paracetamol determinant [12, 13].

Ferric chloride, Folin-Ciocalteu reagent, Nessler's reagent, and Lieberman reagent are some of the colorimetric reagents for paracetamol detection [14]. One of the potential substrate materials uses polymer. The polymer-based substrate has been widely utilized in the field of water treatment, pharmacology, and biology [15]. It has many advantages for a lab-on-chip device, since it is simple and cost-effective, and it also has disposal material [16].

In this experiment, an optical sensor membrane has been developing for paracetamol determination based on colorimetric reaction. Poly(methyl methacrylate) (PMMA) is chosen as membrane material due to its high thermal stability, mechanical strength, and chemical inertness [17]. The specific colorimetric reagent was mixed into a polymer to react with paracetamol. A phase inversion method, as a straightforward and rapid fabrication method, was applied to synthesize polymer membrane [18]. The interfering ion and application of this optical sensor membrane to herbal medicine were also investigated. The result shows that this system applies to paracetamol detection in herbal medicine and is comparable to instrument method.

## 2. Materials and Methods

All chemicals used were of analytical grade, and they were used without further purification. All solutions were made using distilled water. Acetosal, ferric chloride (FeCl_3_), folin, ibuprofen, potassium iodide (KI), mercury chloride (HgCl_2_), sodium hydroxide (NaOH), sodium nitrite (NaNO_2_), and paracetamol were purchased from Merck. Sulphuric acid (H_2_SO_4_) and ethanol 96% were obtained from Emsure. Metampyrone was obtained from Medialabs. Ethyl acetate was obtained from Bratacho Chemistry. Poly(methyl methacrylate) (PMMA) was obtained from Aldrich Chemistry. The characteristic of polymer membrane was observed by Scanning Electron Microscope-Energy Dispersive X-Ray (SEM-EDX) (Jeol JSM-651OLA). The absorbance measurement was recorded by UV-Visible spectrophotometer (Analytic Jena Specord 200).

### 2.1. Reagent Preparation

The reagents used in this experiment are ferric chloride, Folin-Ciocalteu reagent, Nessler's reagent, and Lieberman reagent. Ferric chloride 0.5 M was made in water, whereas Folin-Ciocalteu reagent was made by dissolving 100 g sodium tungstate and 25 g sodium molybdate in 500 ml water, then 50 ml phosphoric acid and 100 ml chloride acid were added and refluxed for 10 hours. The solution is cooled at room temperature, and 150 ml lithium sulfate, 50 ml water, and four drop bromine solution were added and allowed to react for 2 hours. The solution is boiled for 15 minutes, allowed to cool, and then filtered and diluted up to 1000 ml. The reagent was used by diluting it with two portions of water and by adding 4M sodium hydroxide for basic condition. Nessler's reagent was made by adding potassium iodide to the mercury chloride saturated solution. Liebermann reagent was made by dissolving 5 g of sodium nitrite in 50 ml sulphuric acid. These reagents were then tested against paracetamol standards to know its color changes.

### 2.2. Fabrication of Optical Sensor Membrane Using PMMA

PMMA was made in three different concentrations: 5%, 7.5%, and 10%. Each concentration was made by dissolving it in a mixture solvent. The mixture solvent contains ethyl acetate that acts as a solvent and a specific reagent for paracetamol (ferric chloride, Folin-Ciocalteu reagent, Nessler's reagent, and Lieberman reagent). This mixture solvent was made in the different ratio of solvent and reagent, which are 6:4, 7:3, and 8:2, respectively. The mixture solvent was shaken at 100 rpm speed for 5 minutes. Afterwards, the solution was coated on a glass plate and was allowed to dry at room temperature. After the fabrication was complete, the polymer membrane was cut into 1 cm^2^. The polymer membrane was characterized by three steps: characterization of the interaction between optical sensor membrane and paracetamol standards, characterization of optical sensor membrane using Scanning Electron Microscope-Energy Dispersive X-Ray (SEM-EDX), and performance of optical sensor membrane which consists of sensitivity test, stability test, and selectivity test.

### 2.3. Application for Real Sample

A real herbal medicine sample was collected from Bandung, West Java, and Sragen, Central Java, Indonesia. 20 samples of herbal medicine with or without registration number were prepared to determine paracetamol using spectrophotometry UV and optical sensor membrane. The samples (20 g) were extracted using 150 ml ethanol 96% by soxhlation method. The solvent was removed by evaporation, and the residue was dissolved in methanol. The absorbance spectra were recorded at 200–350 nm. For the optical sensor membrane analysis, 3 g of sample was dissolved in 10 ml ethanol 96% and then the sample was added to the membrane.

## 3. Results and Discussion

### 3.1. Reagent Preparation and Characterization of Interaction between Colorimetric Reagent and Paracetamol

The colorimetric sensing ability of reagent of paracetamol (ferric chloride, Folin-Ciocalteu reagent, Nessler's reagent, and Lieberman reagent) was investigated by adding paracetamol to the reagent solution.

Table 1 shows that the solution color changed after paracetamol was added to the FeCl_3_, Folin-Ciocalteu, and Nessler's reagent. FeCl_3_ gives blue and black colors, and the Folin-Ciocalteu gives a dark blue color when this reagent is reacted with phenol on paracetamol. Nessler reagent is aliphatic amide detection and provides an orange-brown colloid when paracetamol is added. Sodium hydroxide solution is used to stabilize the complexity of the reaction between paracetamol and reagent. The color of Lieberman reagent solution will change from colorless to violet if it is heated and is added to a single substitute benzene [14]. In this experiment, the heating procedure is avoided, because it is not applicable to optical sensor membrane. As a result, the color of Lieberman reagent did not change, so this reagent did not continue to be used in optical membrane sensor.

### 3.2. Characterization of Interaction between Optical Sensor Membrane and Paracetamol

Poly(methyl methacrylate) (PMMA) is a highly weather-resistant polymer and it is optically transparent [19]. The chemical inertness of PMMA makes it a good candidate to be applied in optical sensor membrane. The critical point in this fabrication is choosing the right solvent that can dissolve the PMMA and the reagent. All of the specific reagents for paracetamol (ferric chloride, Folin-Ciocalteu reagent, and Nessler's reagent) are water soluble. The solubility of PMMA can be predicted by Hildebrand solubility parameter. If the polymer and solvent have similar Hildebrand solubility value, the polymer will be easily soluble in that solvent [20]. The Hildebrand solubility value of PMMA is 9.3 kal^1/2^ cm^−3/2^ [21]. The solvent that has similar value and that is possible to dissolve the reagent is ethyl acetate with Hildebrand solubility value of 9.1 kal^1/2^ cm^−3/2^ [22]. Then, ethyl acetate is used as a solvent to dissolve the PMMA and the reagent. Based on a preliminary study (data were not shown), the concentration of PMMA that is below 5% produces a fragile membrane, whereas the concentration that is above 10% produces a dense membrane and it makes it difficult to absorb the reagent. Therefore, the PMMA was prepared in the concentration of 5%, 7.5%, and 10% of the ratio of the solvent and reagent is 6:4, 7:3, and 8:2 for each concentration. After the fabrication was complete, 100 mg/ml of paracetamol solution was added to the membrane and was allowed to react with the reagent.

Table 2 shows that all of the variations of optical sensor membrane give a positive result with different reaction time. This reaction time is affected by the size of the pore on the membrane. The response time increased along with the increase of the concentration of the polymer. In this system, when the concentration of the polymer increases, the concentration of the reagent decreases. The high concentration of polymer makes the membrane more dense [23] and the sample needs more time to adsorb and react with the small concentration of reagent in the membrane. The optical sensor membrane of PMMA-FeCl_3_ gives a positive result with the shortest time reaction. The best performance of this sensor is 7.5% PMMA with mixture solvent ratio of 7:3.

As shown in Table 3, the color of this sensor changed from yellow to blue when it reacted with paracetamol. The color of sensor membrane of PMMA-Folin-Ciocalteu changed from green to dark blue in basic condition and from white to brown for PMMA-Nessler, with the best result of 5% PMMA in 6:4 mixture solvent for both. When all of these membranes (PMMA-FeCl_3_; PMMA-Folin-Ciocalteu; PMMA-Nessler) give the positive result, the sample contains paracetamol.

### 3.3. Characterization of Optical Sensor Membrane Using Scanning Electron Microscope-Energy Dispersive X-Ray (SEM-EDX)

**Figure 1:**
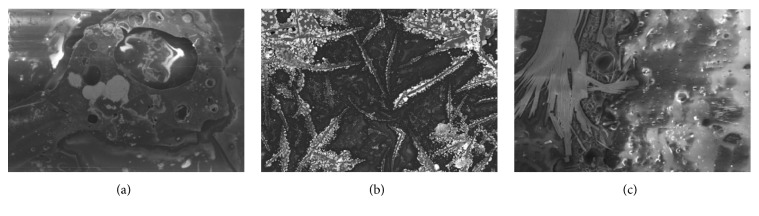
Morphology of the optical sensor membrane by SEM-EDX: (a) PMMA-FeCl_3_ 7.5% 7:3; (b) PMMA-Folin-Ciocalteu 5% 6:4; (c) PMMA-Nessler 5% 6:4.

The best result of optical sensor membrane was characterized by Scanning Electron Microscope-Energy Dispersive X-Ray (SEM-EDX) to observe the morphology of membrane and to confirm the element on the membrane. Visually, all of these membranes are homogeneous (Table 2). As shown in Figure 1, the optical sensor membrane of PMMA-FeCl_3_ has the smallest pore and is more homogenous compared to the others. It causes this membrane to have the shortest time reaction. In the following, Table 4 shows that the elements present in the membrane are the same as the constituent element. This result confirms that the reagent is absorbed into the membrane and it reacts with paracetamol.

### 3.4. Performance of Optical Sensor Membrane

#### 3.4.1. Sensitivity Test

The sensitivity level of optical sensors membrane was analyzed by determining the lowest measurable concentration of paracetamol that can be detected. In this experiment, different amounts of paracetamol (0 – 20 mg/ml) were added to the membrane. The time reaction of the color change in the presence of paracetamol was also observed. The design of optical sensor membrane for paracetamol detection will contain three strips of PMMA-FeCl_3_; PMMA-Folin-Ciocalteu; PMMA-Nessler.  Table 5 shows that the result is in accordance with the minimum dosage form of paracetamol usually added to herbal medicine at 500 mg/3000 mg. The different lowest concentration and reaction time of the membrane were influenced by a particular reagent. This result corresponds to Table 2 that FeCl_3_ has the shortest response time. The intensity of the color change increased along with the increase of paracetamol concentration.

#### 3.4.2. Stability Test

In this experiment, the optical sensor membrane was stored in a zip-lock plastic at room temperature. The membranes were tested by reacting with paracetamol daily until the color ceased to change when paracetamol was added. The results are summarized in Table 6.

#### 3.4.3. Selectivity Test

In addition to paracetamol, metampyrone, acetosal, and ibuprofen were usually added to herbal medicine because they have the same therapeutic effect. Chemically, all of these drugs have a similar structure to paracetamol. The selectivity of the membrane was evaluated by testing the membrane with these chemical drugs. Metampyrone, acetosal, and ibuprofen were dissolved in ethanol (100 mg/ml) and were then added to the membrane, respectively. As shown in Table 7, the color of PMMA-FeCl_3_ changed to brown and purple when metampyrone and acetosal were added, respectively. This result does not affect the determination of paracetamol since the color of the membrane in the presence of paracetamol is blue. The PMMA-Folin-Ciocalteu membrane gives the same color when paracetamol and metampyrone were added. To show that paracetamol is a presence in the sample, the three membranes must give the positive result. Although the PMMA-Folin-Ciocalteu membrane gives the same color when metampyrone was added, a different color is obtained on PMMA-FeCl_3_ and no color change is observed on PMMA-Nessler. This result is technically applicable and it does not break government regulation since the regulation states that herbal medicine should not contain the chemical drug.

#### 3.4.4. Application for Real Sample

To demonstrate the applicability of optical sensor membrane for real sample analysis, 20 herbal medicine samples were analyzed using spectrophotometry UV and optical sensor membrane. Paracetamol was not detectable in these samples by using the developed optical sensor membrane and spectrophotometry UV. This can show that the sample which contains herbal medicine does not have an effect on the paracetamol detection. The sample was then spiked with 4.5 mg/ml of paracetamol. The results show good agreement between optical sensor membrane method and spectrophotometry data. Both methods confirm that the sample contains paracetamol. The reaction time of the membrane to detect paracetamol is summarized in Table 8.

## 4. Conclusions

The poly(methyl methacrylate) (PMMA) is a polymer that can be developed as optical sensor membrane. This membrane will absorb specific colorimetric reagent for paracetamol detection, which makes it applicable for paracetamol detection in herbal medicine. The characteristic of this membrane depends on the reagent and the concentration of polymer and solvent that are mixed into the membrane. The best results of optical sensor membrane are PMMA-FeCl_3_ 7.5% (7:3), PMMA-Folin 5% (6:4), and PMMA-Nessler 5% (6:4). In general, all of the optical sensor membranes are simple and applicable for on-site paracetamol analysis in herbal medicine.

## Figures and Tables

**Table 1:** Color change of the reagent in the presence of paracetamol (100 mg/ml).

Reagent	Reagent solution	Blank	Added paracetamol
FeCl_3_	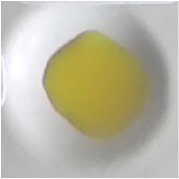	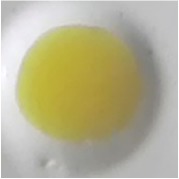	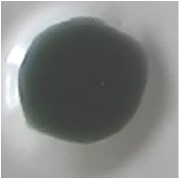
Folin-ciocalteu	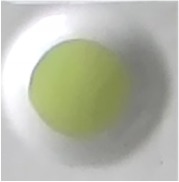	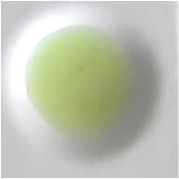	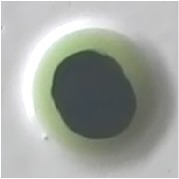
Nessler's reagent	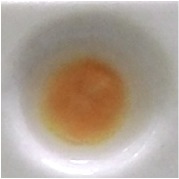	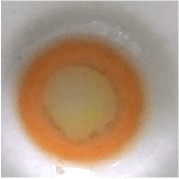	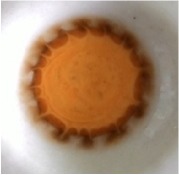
Lieberman	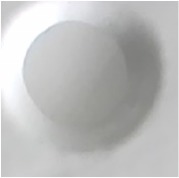	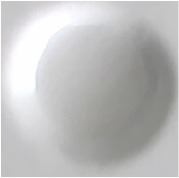	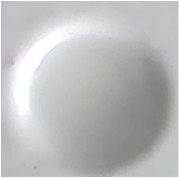

Blank: reagent solution + ethanol as a paracetamol solvent.

**Table 2:** Characterization of optical sensor membrane in the presence of paracetamol.

Reagent	%PMMA	Mixture Solvent (EA:Reagent)	Homogeneous polymer	Result	Reaction time
FeCl_3_	5%	6:4	Non-homogeneous	+	1”
		7:3	Non-homogeneous	+	1”
		8:2	Non-homogeneous, fragile	+	1”
	7.5%	6:4	Homogeneous, fragile	+	1”
		7:3	Homogeneous	+	1”
		8:2	Homogeneous	+	1”
	10%	6:4	Non-homogeneous	+	1”
		7:3	Homogeneous	+	1”
		8:2	Non-homogeneous	+	1”

Folin-Ciocalteu	5%	6:4	Homogeneous	+	5”
		7:3	Homogeneous	+	11”
		8:2	Homogeneous	+	10”
	7.5%	6:4	Homogeneous	+	7”
		7:3	Homogeneous	+	10”
		8:2	Homogeneous	+	41”
	10%	6:4	Homogeneous	+	45”
		7:3	Homogeneous	+	50”
		8:2	Homogeneous	+	1'31”

Nessler's	5%	6:4	Homogeneous	+	7”
		7:3	Homogeneous	+	12”
		8:2	Homogeneous	+	1'37”
	7.5%	6:4	Non-homogeneous	+	11”
		7:3	Non-homogeneous	+	32”
		8:2	Non-homogeneous	+	8'40”
	10%	6:4	Homogeneous	+	12”
		7:3	Homogeneous	+	55”
		8:2	Homogeneous	+	6'30”

EA: ethyl acetate; (+): suitable color changes; (‘): minute; (“): second.

**Table 3:** Color test of optical sensor membrane in the presence of paracetamol.

Optical sensor membrane	Membrane	Added paracetamol
PMMA-FeCl_3_ 7.5% 7:3	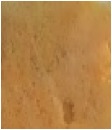	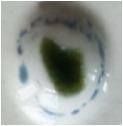
PMMA-Folin-Ciocalteu 5% 6:4	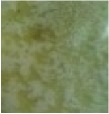	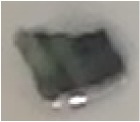
PMMA-Nessler's reagent 5% 6:4	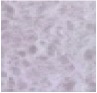	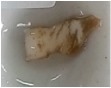

**Table 4:** The elemental content of the membrane.

Membrane	Element	Element concentration (%)
PMMA-FeCl_3_ 7.5% 7:3	Carbon	70.944
	Oxygen	26.268
	Chlorine	1.846
	Iron	0.942

PMMA-Folin-Ciocalteu 5% 6:4	Carbon	59.462
	Oxygen	36.219
	Sodium	1.233
	Phosphor	0.674
	Sulphur	1.432
	Chlorine	0.651
	Tungsten	0.330

PMMA-Nessler's reagent 5% 6:4	Carbon	72.478
	Oxygen	18.102
	Chlorine	5.741
	Potassium	1.074
	Mercury	2.605

**Table 5:** The lowest measurable concentration of paracetamol on the membrane.

Optical sensor membrane	Concentration mg/ml	Reaction time
PMMA- FeCl_3_ 7.5% 7:3	2.55	3' 17”
PMMA-Folin-Ciocalteu 5% 6:4	4.05	5'
PMMA-Nessler 5% 6:4	4.01	2' 50”

**Table 6:** The stability time of optical sensor membrane.

Optical sensor membrane	Stability time (day)
PMMA-FeCl_3_ 7.5% 7:3	122
PMMA-Folin-Ciocalteu 5% 6:4	125
PMMA-Nessler 5% 6:4	125

**Table 7:** The robustness test of optical sensor membrane.

Optical sensor membrane	Metampyrone		Acetosal		Ibuprofen	
	Color change	Reaction time	Color change	Reaction time	Color change	Reaction time
PMMA-FeCl_3_ 7.5% 7:3	Brown	1”	Purple	1”	-	-
PMMA-Folin-Ciocalteu 5% 6:4	Dark blue	3' 57”	-	-	-	-
PMMA-Nessler 5% 6:4	-	-	-	-	-	-

**Table 8:** Reaction time of optical sensor membrane on detection of paracetamol in herbal medicine sample.

Sample	Reaction time		
	PMMA-FeCl_3_ 7,5% 7:3	PMMA-Folin 5% 6:4	PMMA-Nessler 5% 6:4
1	5'45”	8'20”	11'25”
2	5'	8'10”	11'40”
3	5'25”	8'45”	11'05”
4	5'06”	8'30”	12'05”
5	5'37”	9'03”	11'45”
6	5'20”	8'50”	11'59”
7	6'01”	8'16”	11'45”
8	5'55”	8'34”	11'39”
9	5'05”	8'25”	11'46”
10	5'10”	8'43”	11'49”
11	5'30”	9'	12'07”
12	5'45”	8'12”	13'03”
13	6'06”	8'05”	11'55”
14	5'19”	8'56”	11'53”
15	5'05”	8'03”	11'57”
16	5'10”	7'58”	12'07”
17	5'58”	8'27”	11'55”
18	5'07”	8'50”	11'59”
19	5'29”	8'15”	11'45”
20	5'48”	8'28”	11'58”

## Data Availability

The data used to support the findings of this study are included within the article.
